# The determinants of COVID-19 vaccination intention: a meta-review

**DOI:** 10.3389/fpubh.2023.1162861

**Published:** 2023-06-12

**Authors:** Yam B. Limbu, Rajesh K. Gautam

**Affiliations:** ^1^Montclair State University, Montclair, NJ, United States; ^2^Dr. Hari Singh Gour University, Sagar, Madhya Pradesh, India

**Keywords:** COVID-19, vaccination intention, meta-review, systematic review of systematic reviews, COVID-19 vaccine

## Abstract

**Background:**

A large number of systematic reviews have been published that synthesized various determinants of COVID-19 vaccination intention (CVI). However, they reported inconsistent evidence. Therefore, we conducted a meta-review (systematic review of systematic reviews) to provide a comprehensive synthesis of factors influencing CVI.

**Methods:**

This meta-review was conducted in accordance with PRISMA guidelines. PubMed, Scopus, Web of Science, and CINAHL were searched for systematic reviews published from 2020 to 2022 that examined the determinants of CVI. AMSTAR-2 critical appraisal tool was used to ensure the quality of included reviews, and ROBIS tool was used to evaluate the risk of bias.

**Results:**

Globally, the average rate of COVID-19 vaccination intention was 56.97%. We identified 21 main determinants of CVI: socio-demographic, geographical location, social, political, government role, study timeline, attitude, perceived severity, perceived susceptibility, perceived benefits, perceived barriers, self-efficacy and perceived behavioral control, norms, trust, conspiracy theory/propaganda/misinformation, knowledge, information and communication, vaccination recommendation, vaccination history, history of COVID-19 infection, and health status and well-being.

**Conclusions:**

These results suggest that COVID-19 vaccination intention is a complex process and is affected by numerous multidimensional factors. Therefore, integrated communication strategies and multifaceted interventions may be effective for improving vaccination intention against COVID-19.

## 1. Introduction

Several pandemics have been recorded in history, but the emergence of SARS-CoV-2 in the latter part of 2019 is one of the deadliest public health crises in our living memory (1). On January 30, 2020, the World Health Organization (WHO) declared it a Public Health Emergency of International Concern (PHEIC). This declaration was made in response to the rapid spread of the virus beyond China. Various restriction measures were imposed throughout the world to restrain the spread of the virus. As it was a novel Coronavirus, efforts were made to discover its treatment and invent vaccines to combat its challenges. Several vaccines were developed, and as of May 9, 2023, more than 13 billion vaccine doses have been administered. As per WHO Coronavirus Dashboard (2) on May 9, 2023, globally, more than 765 million confirmed cases and 6.9 million deaths were reported. On May 4, 2023, in the fifteenth meeting of the International Health Regulations (2005) Emergency Committee of WHO, it was declared that the ongoing COVID-19 pandemics now an established and ongoing health issue that no longer constitutes a PHEIC. The Committee highlighted the decreasing trend of hospitalization and death due to COVID-19 and the high levels of population immunity to SARS-CoV-2. WHO also emphasized that the virus remains a global health threat as it continues to spread and its new variants are expected to continue to emerge (3).

However, a significant proportion of the world's population is still unvaccinated, posing a continuous public health concern. Some populations, especially women, single, young adults, patients, and healthcare workers, are still hesitant to get primary series or boosters (4–7). Likewise, increasing vaccination rates, especially booster vaccination among specific groups, such as children, was an immense obstacle in some countries, such as Jordan (8), Croatia (9), and China (10). Therefore, to manage COVID-19 and control its new variants, continuous efforts should be made by governments and international health agencies to overcome misperceptions about the virus. Furthermore, in light of the WHO's recent PHEIC declaration for COVID-19 and declining confirmed cases and deaths, vaccination promotion campaigns should not only focus on highlighting the benefits of vaccines and the severity and susceptibility of the virus but also identify the factors that influence public's continuous support for COVID-19 vaccination.

In this study, COVID vaccination intention (CVI) refers to the willingness to be vaccinated, vaccine acceptability including desirability, vaccine demand, and positive attitudes toward the given vaccine, which is contrasted to vaccine hesitancy, the delay or refusal to be vaccinated (11). There are numerous barriers to vaccination campaigns, even when the vaccines are freely accessible or affordable. Some barriers reported by previous studies include psychological (12, 13), socio-economic (14–17), and demographic (18–20).

Numerous systematic reviews, scoping reviews, rapid reviews, and meta-analyses have been published from different parts of the world with the coverage of diverse populations and regions on vaccination intention. However, they reported inconsistent findings with regard to the drivers influencing vaccination acceptance and vaccination intention rates (4, 21–23). Hence, the objective of this meta-review (systematic review of systematic reviews) is to provide a comprehensive overview of existing evidence on factors influencing the COVID-19 vaccination intention published by different types of review and to offer some avenues for future research. More specifically, the present study contributes literature in several ways. First, to our knowledge, this is the first study to systematically map and synthesize key findings of the systematic reviews and identify major factors driving COVID-19 vaccination acceptance. Secondly, this meta-review included different types of reviews including systematic reviews with meta-analyses, scoping reviews, rapid reviews, and systematic review with no meta-analyses for a broader and a holistic understanding about vaccination intention and its determinants reported around the globe. Thirdly, this meta-review provides directions for future research. Finally, this will report an overall global vaccination intention rate and vaccine acceptance across geographic locations.

## 2. Methodology

A meta-review requires a critical appraisal of the methodological quality of systematic reviews and meta-analyses. For this review, the guidelines of Preferred Reporting Items for Systematic Reviews and Meta-Analyses (PRISMA) were followed (24, 25). ROBIS (Risk of Bias in Systematic Reviews) tool (26) was used to assess the bias in the search, selection, data extraction, and synthesis. AMSTAR-2 critical appraisal tool (27) was used to ensure the methodological quality of systematic reviews included in this meta-review. In this study, the term ‘systematic reviews' refers to different types of reviews, including systematic reviews with or without meta-analyses, scoping reviews, mapping reviews, literature reviews, and rapid reviews.

### 2.1. Search strategy

We conducted a comprehensive search of published literature from four databases (PubMed, Web of Science, CINAHL, and Scopus) using various keywords, such as “review”, “vaccination intention”, and “COVID-19”, “coronavirus”, or “SARS-CoV-2”. The combinations of search terms and Boolean operators that were used to locate studies in each database are presented in Table 1.

**Table 1:** Search strategy.

**Searched database**	**Search terms and Boolean operators**	**No. of records**
Scopus	TITLE (review) AND ALL (vaccination AND intention) OR (vaccine AND acceptance) AND ALL (covid-19) OR (coronavirus) OR (SARS-CoV-2) AND (LIMIT-TO (PUBYEAR, 2022) OR LIMIT-TO (PUBYEAR, 2021) OR LIMIT-TO (PUBYEAR, 2020)) AND (LIMIT-TO (DOCTYPE, “re”)) AND (LIMIT-TO (LANGUAGE, “English”)) AND (LIMIT-TO (SRCTYPE, “j”))	367
Web of Science	(((TI=(review)) AND ALL=(vaccination intention)) OR ALL=(vaccine acceptance)) AND TI=(covid-19) and Review Article (Document Types) and 2020 or 2021 or 2022 (Publication Years) (((TI=(review)) AND ALL=(vaccination intention)) OR ALL=(vaccine acceptance)) AND TI=(coronavirus) and Review Article (Document Types) and 2020 or 2021 or 2022 (Publication Years) (((TI=(review)) AND ALL=(vaccination intention)) OR ALL=(vaccine acceptance)) AND TI=(SARS-CoV-2) and Review Article (Document Types) and 2020 or 2021 or 2022 (Publication Years)	147
PubMed	(((review[Title]) AND (vaccination intention) OR (vaccine acceptance)) AND (covid-19) (((review[Title]) AND (vaccination intention) OR (vaccine acceptance)) AND (coronavirus) (((review[Title]) AND (vaccination intention) OR (vaccine acceptance)) AND (SARS-CoV-2)	197
CINAHL	TI review AND TX vaccination intention OR TX vaccine acceptance AND TI covid-19 TI review AND TX vaccination intention OR TX vaccine acceptance AND TI coronavirus TI review AND TX vaccination intention OR TX vaccine acceptance AND TI SARS-CoV-2	366

**Figure 1:**
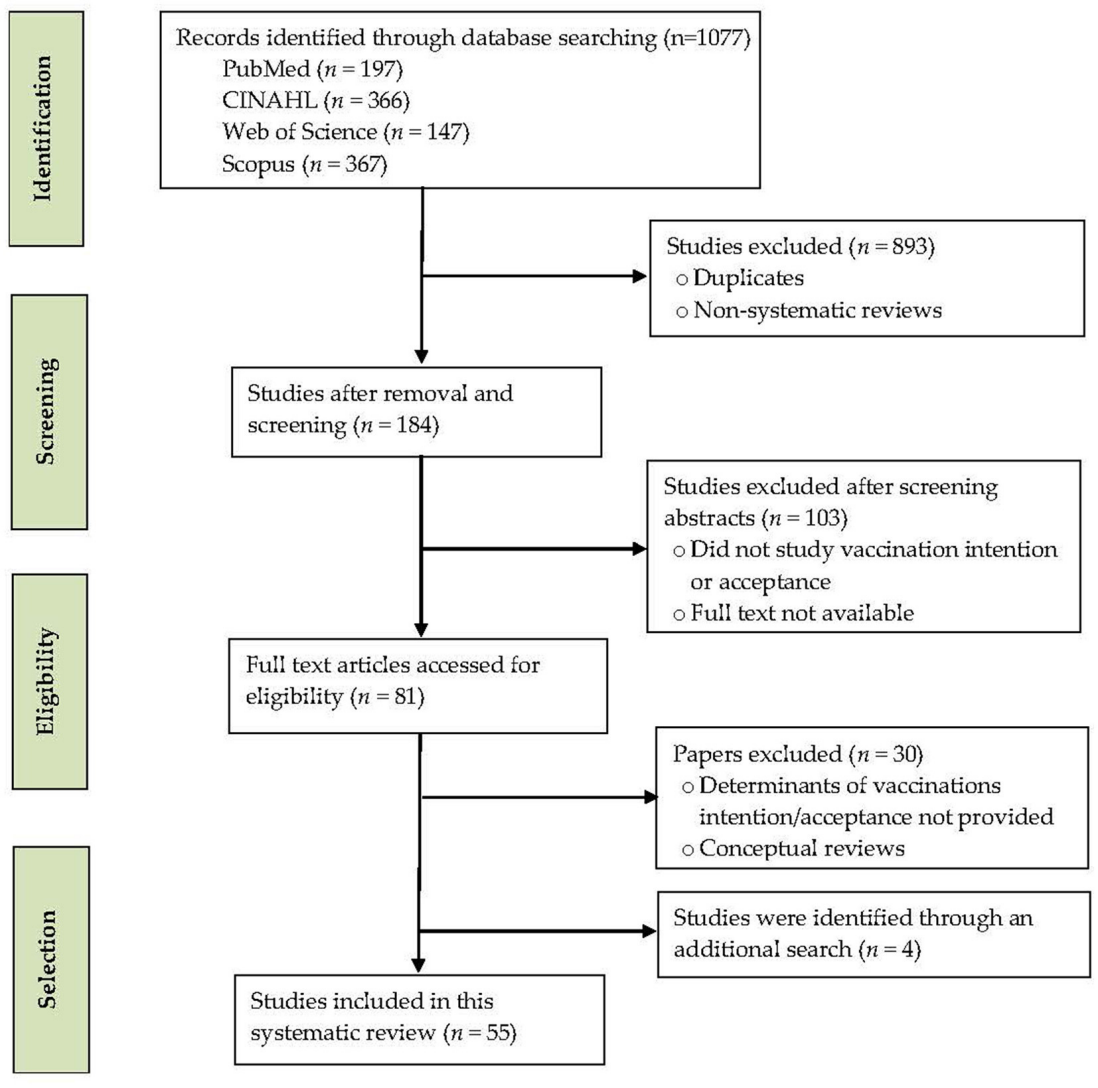
PRISMA flow diagram of study selection.

To demonstrate the study selection process, the number of records identified, screened, and excluded, and the reasons for exclusion, a PRISMA flow diagram is drawn (Figure 1). A total of 1077 records were retrieved from the databases. Of them, 893 records were removed for duplicates, non-systematic reviews, and non-peer-reviewed reviews. A total of 103 records were excluded after screening the abstracts that were irrelevant or did not study vaccination intention and its determinants. The remaining 81 full-text systematic reviews were further assessed for eligibility. Furthermore, four eligible systematic reviews were identified through an additional search. Fifty-five full-length reviews published from January 2020 to December 2022 were retrieved for this meta-review.

### 2.2. Inclusion and exclusion criteria

The following inclusion and exclusion criteria were used to identify relevant systematic reviews.

#### 2.2.1. Inclusion criteria

- Systematic reviews that reported the predictors of CVI.
- Systematic reviews published in peer-reviewed journals.
- Systematic reviews published in English.

#### 2.2.2. Exclusion criteria

- Systematic reviews that reported the determinants of COVID-19 vaccine hesitancy.
- Qualitative/narrative reviews.
- Non-systematic reviews.
- Non-peer-reviewed systematic reviews.

Two researchers independently screened the titles and abstracts of the identified systematic reviews. Full-text systematic reviews were obtained whose titles and abstracts met inclusion criteria. All full-text systematic reviews were then evaluated to confirm if they reported necessary information or statistics on vaccination intention with respect to COVID-19.

### 2.3. Risk of bias

To ensure the methodological quality and risk of bias, ROBIS tool was used as per the guidelines of Whiting et al. (26). To evaluate the level of bias present in a systematic review and to assess specific concerns about potential biases in the search, selection, data extraction, and synthesis, ratings were used to judge the overall risk of bias. The signaling questions were answered as “yes”, “probably yes”, “probably no”, “no”, or “no information”. The subsequent level of concern about bias associated with each domain was then judged as “low”, “high”, or “unclear”. If the answers to all signaling questions for a domain were “yes” or “probably yes”, the level of concern was judged as low. If any signaling question was answered “no” or “probably no”, then a bias existed. Two researchers independently used the ROBIS tool to perform risk of bias and to identify eligible systematic reviews to be included in the present meta-review. Any disagreements were resolved through discussion or a decision made by an expert, a third umpire. Similarly, the selection of databases or digital libraries was also decided with consensus.

### 2.4. Critical appraisal of included reviews

A critical appraisal of included reviews was conducted using the tool AMSTAR-2 (27) and displayed in Table 2. It was noticed that a few reviews did not meet some criteria. However, most studies complied with a large number of criteria. All the included reviews fulfilled some criteria, such as 1, 2, 5, 6, and 14. They were marked by a + sign or “yes”. Criterion 1 is about the components of PICO (population, intervention, control group, and outcome), whether the included reviews have details of PICO or not. We found that all reviews met this criterion. Similarly, all the reviews also complied with criteria 2, 5, 6, and 14. Criterion 6 is about unbiased data extraction, and we found that data extraction of all included reviews was unbiased; similarly, criterion 14 (the discussion of heterogeneity) was observed in the results.

**Table 2:** Results of critical appraisal of included reviews.

**Author(s)**	**Year**	**1**	**2**	**3**	**4**	**5**	**6**	**7**	**8**	**9**	**10**	**11**	**12**	**13**	**14**	**15**	**16**
Abdelmoneim et al. (28)	2022	+	+	+	+	+	+	+	+	+	+	+	+	+	+	+	+
Ackah et al. (29)	2022	+	+	‡	–	+	+	+	+	+	+	+	+	+	+	+	+
Al-Amer et al. (1)	2022	+	+	+	+	+	+	+	+	+	‡	–	–	+	+	+	+
Alarcón-Braga et al. (23)	2022	+	+	+	+	+	+	+	+	+	+	+	+	+	+	+	+
Alemayehu et al. (30)	2022	+	+	+	+	+	+	+	+	+	+	+	+	+	+	+	+
Alimohamadi et al. (31)	2022	+	+	+	+	+	+	+	+	+	‡	+	+	+	+	+	+
Al-Jayyousi et al. (32)	2021	+	+	+	+	+	+	+	+	–	–	–	–	–	+	–	+
Azami et al. (33)	2022	+	+	+	+	+	+	+	+	‡	+	+	+	‡	+	‡	+
AlShurman et al. (34)	2021	+	+	+	+	+	+	‡	+	‡	+	–	–	‡	+	‡	+
Bayou and Amare (35)	2022	+	+	‡	+	+	+	+	+	‡	+	–	–	‡	+	‡	+
Belay et al. (36)	2022	+	+	+	+	+	+	+	+	+	+	+	+	+	+	+	+
Bhattacharya et al. (37)	2022	+	+	+	+	+	+	+	‡	+	+	+	+	+	+	+	+
Biswas et al. (38)	2021	+	+	+	+	+	+	+	+	‡	+	–	–	‡	+	‡	+
Chen et al. (39)	2022	+	+	+	+	+	+	‡	+	‡	+	+	+	‡	+	‡	+
Desye (40)	2022	+	+	+	+	+	+	+	+	‡	‡	–	–	‡	+	‡	+
Galanis et al. (41)	2021	+	+	+	+	+	+	+	+	‡	–	+	+	‡	+	‡	+
Galanis et al. (42)	2022	+	+	+	+	+	+	+	+	+	+	+	+	+	+	+	+
Galanis et al. (43)	2022	+	+	+	+	+	+	+	+	+	+	+	+	+	+	+	+
Geng et al. (44)	2022	+	+	+	+	+	+	+	+	‡	+	+	+	‡	+	‡	+
Halemani et al. (45)	2022	+	+	+	+	+	+	+	+	+	+	+	+	+	+	+	+
Hajure et al. (46)	2021	+	+	+	+	+	+	+	+	‡	+	–	–	‡	+	‡	+
Januszek et al. (47)	2021	+	+	+	–	+	+	+	+	‡	+	–	–	‡	+	‡	+
Joshi et al. (48)	2021	+	+	‡	–	+	+	+	+	‡	–	–	–	‡	+	‡	+
Kalu et al. (37)	2022	+	+	+	+	+	+	–	+	‡	–	–	–	‡	+	‡	–
Kamal et al. (49)	2021	+	+	+	+	+	+	+	+	+	+	–	–	+	+	+	+
Kazeminia et al. (50)	2022	+	+	+	+	+	+	+	+	+	+	+	+	+	+	+	+
Kukreti et al. (51)	2022	+	+	+	+	+	+	+	+	+	+	+	+	+	+	+	+
Li et al. (52)	2021	+	+	+	+	+	+	+	+	+	+	–	–	+	+	+	+
Limbu et al. (21)	2022	+	+	+	+	+	+	+	+	–	+	+	+	–	+	–	+
Lin et al. (53)	2021	+	+	+	+	+	+	+	+	–	+	–	–	–	+	–	+
Lin et al. (54)	2022	+	+	+	+	+	+	+	+	+	+	+	+	+	+	+	+
Luo et al. (55)	2021	+	+	+	+	+	+	+	+	‡	+	+	+	‡	+	‡	+
Mahmud et al. (56)	2022	+	+	+	+	+	+	+	+	‡	+	+	+	‡	+	‡	+
Mose et al. (57)	2022	+	+	+	+	+	+	+	+	+	+	+	+	+	+	+	+
Nehal et al. (58)	2021	+	+	‡	–	+	+	+	+	+	+	+	+	+	+	+	+
Nindrea et al. (59)	2021	+	+	+	+	+	+	+	+	‡	+	+	+	‡	+	‡	–
Norhayati et al. (60)	2022	+	+	+	+	+	+	+	+	+	–	+	+	+	+	+	+
Olu-Abiodun et al. (61)	2022	+	+	+	+	+	+	+	+	‡	+	–	–	‡	+	‡	+
Parthasarathi et al. (62)	2022	+	+	+	+	+	+	+	+	+	–	+	+	+	+	+	+
Patwary et al. (63)	2022	+	+	+	+	+	+	+	+	+	+	+	+	+	+	+	+
Popa et al. (64)	2022	+	+	+	‡	+	+	+	+	–	+	–	–	–	+	–	+
Prabani et al. (65)	2022	+	+	+	+	+	+	+	+	+	+	+	+	+	+	+	+
Ripp and Roer (66)	2022	+	+	‡	+	+	+	+	+	‡	+	–	–	‡	+	‡	+
Robinson et al. (67)	2021	+	+	+	+	+	+	+	+	+	+	+	+	+	+	+	+
Roy et al. (68)	2022	+	+	‡	+	+	+	+	+	‡	+	–	–	‡	+	‡	+
Sahile et al. (69)	2022	+	+	+	+	+	+	+	+	+	‡	+	+	+	+	+	+
Shakeel et al. (70)	2022	+	+	+	+	+	+	+	+	‡	+	–	–	‡	+	‡	+
Shamshirsaz et al. (71)	2022	+	+	+	+	+	+	+	+	+	‡	+	+	+	+	+	+
Shui et al. (72)	2022	+	+	+	+	+	+	+	+	+	+	+	+	+	+	+	+
Terry et al. (73)	2022	+	+	+	+	+	+	+	+	+	+	+	+	+	+	+	+
Wake (74)	2021	+	+	‡	+	+	+	+	+	‡	–	–	–	‡	+	‡	+
Wang et al. (75)	2021	+	+	+	+	+	+	+	+	‡	+	+	+	‡	+	‡	+
Wang et al. (76)	2022	+	+	+	+	+	+	+	+	+	+	+	+	+	+	+	+
Willems et al. (77)	2022	+	+	‡	+	+	+	+	+	‡	+	–	–	‡	+	‡	+
Zintel et al. (78)	2022	+	+	+	+	+	+	+	+	‡	+	+	+	‡	+	‡	+

+ for “yes”; – for “no” and ‡ for partial “yes” or “unclear”.

The highest number of negative responses, i.e., “no” or—sign, was recorded for criteria 11 and 12. Out of 55 reviews included in the present study, approximately one-third, i.e., 20 reviews, did not perform a meta-analysis. These reviews also did not comply with criterion 12. Partial “yes” or “unclear” or ‡ sign was also recorded. The highest responses were recorded for criteria 9, 13, and 15. Five out of 55 reviews did not meet criterion 4 (29, 47, 48, 58, 64). These reviews were based on a search of a single database; the remaining searched two or more databases.

Overall, we found that the vast majority of the reviews satisfied most of the necessary AMSTAR-2 criteria. However, in many cases, not meeting the criteria was due to the fact that there was no mention of the element in the review or it was not stated explicitly enough for the reader to comprehend. There are several reasons for this, such as publishing guidelines of the specific journal, word limitation, different standards of different journals, and the requirement topic chosen for review.

### 2.5. Data extraction and analysis

Data extraction was also performed by the same two researchers independently. The main information that were extracted from studies included author's name, publication year, type of systematic review, vaccination intention rate (%), searched databases, study objective, participants (study population), number of studies included, and determinants of CVI. IBM SPSS Statistics 27 was used to analyze the data.

## 3. Results

### 3.1. Description of included systematic reviews

As presented in Tables 3, 4, the majority of the systematic reviews (70.91%) included in this meta-review were published in 2022, and the remaining were published in 2021. Most reviews (61.82%) were systematic reviews with meta-analyses, followed by systematic reviews with no meta-analyses, rapid reviews, scoping reviews, literature reviews, and mapping reviews. The most frequently searched database was PubMed (54/55), followed by Web of Science (32/55), Scopus (23/55), Embase (19/55), Google Scholar (19/55), Cochrane Library (11/55), Science Direct (11/55), CINAHL (11/55), MEDLINE (9/55), PsycINFO (8/55), and EBSCO (7/55). Twenty-one reviews focused on the general adult population, healthcare workers (13/55), and pregnant women (5/55). The studies included in this meta-review consisted of 2,519 studies conducted across the globe, with an average study of 46.65 (standard deviation = 72.4), ranging from 9 (47) to 519 (76). The systematic review and meta-analysis included the highest average number of studies (50), followed by scoping review (44), systematic review (35), and rapid review (34).

**Table 3:** Characteristics of included systematic reviews.

**Characteristics**		**Frequency**	**Percent**
Publication by year	2022	39	70.91
	2021	16	29.09
Types of review	Systematic review and meta-analysis	34	61.82
	Systematic review	8	14.55
	Rapid review	6	10.91
	Scoping review	5	9.1
	Literature review	1	1.82
	Mapping review	1	1.82
Study population	General population	21	36.21
	Healthcare worker	13	22.41
	Pregnant women	5	8.62
	Ethiopian	4	6.9
	Other African countries	3	5.17
	Student	2	3.45
	Parent	2	3.45
	Other	8	13.79
Search database	PubMed	54	21.51
	Web of science	32	12.75
	Scopus	23	9.16
	Embase	19	7.57
	Google scholar	19	7.57
	Cochrane library	11	4.38
	Science direct	11	4.38
	CINAHL	11	4.38
	MEDLINE	9	3.59
	PsycINFO	8	3.19
	EBSCO	7	2.79
	ProQuest	5	2.0
	Other	42	16.73
Average number of studies included	Range 9–519		46.65
Average CVI	Range 46–78		56.97

CVI, COVID-19 vaccination intention.

**Table 4:** Characteristics of included reviews and factors influencing COVID-19 vaccination intention.

**Author(s)**	**Year**	**Review type**	**Vaccine intention rate %**	**Search source/database**	**No. of studies included**	**Population**	**Key factors influencing vaccination intention**
Abdelmoneim et al. (28)	2022	Systematic review and meta-analysis	81	PsycINFO, Scopus, EBSCO, PubMed, ProQuest, SciELO, SAGE, Web of Science, Google Scholar, Science Direct	48	General population	Previous COVID-19 infection (-), having chronic disease, trust in the vaccine effectiveness, region
Ackah et al. (29)	2022	Systematic review and meta-analysis	46	PubMed, Google Scholar, Africa Journal Online	21	HCW in Africa	Region, higher acceptance among HCW, than healthcare students, side effects of the vaccine, vaccine's safety, efficacy and effectiveness, short duration of the clinical trials, COVID-19 infections, limited information, social trust
Al-Amer et al. (1)	2022	Systematic review	27.7–93.3	CINAHL, Cochrane Library, Google Scholar, ProQuest, PsycINFO, PubMed, Scopus	30	General population, HCW	Socio-demographic, perceptions of risk and susceptibility to COVID-19, vaccine attributes, negative information about COVID-19 vaccines in the social media (-), low confidence in the health system (-)
Alarcón-Braga et al. (23)	2022	Systematic review and meta-analysis	78	PubMed, Scopus, Web of Science	19	Latin America and the Caribbean (LAC) population	Fear of adverse effects (-), distrust in local health systems (-), misinformation or fake news shared in social media (-), health-system-related variables, local concerns (economy, virtual education, teleworking, etc.), political issues (purchase of vaccine batches, quarantine isolation measures, vaccination process implementation, etc.), demographic and geographical variables, entrenched vaccination culture in LAC population, the promotion of the importance of vaccination at the first level of care
Alemayehu et al. (30)	2022	Systematic review and meta-analysis	60.2	PubMed, Google Scholar, Global Health		East Africa	Attending above secondary school, having good knowledge about the vaccine, having a positive attitude toward vaccine, history of COVID-19 infection, male
Alimohamadi et al. (31)	2022	Systematic review and meta-analysis	65.1	PubMed, Scopus, Web of Science	74	General population	HCWs (-) vs. general population, region Middle East (-) vs. South America
Al-Jayyousi et al. (32)	2021	Scoping review	29.4–86.	PubMed, Embase, Web of Science, Cochrane Central	50	General population, HCW	Socio-demographic, individual factors, social and organizational factors, certain characteristics of COVID-19 vaccines
Azami et al. (33)	2022	Systematic review and meta-analysis	53.46	PubMed, Web of Science, Scopus, Science Direct, Cochrane Library, Embase, EBSCO, Google Scholar	16	Pregnant women	Month of the study
AlShurman et al. (34)	2021	Scoping review	60–93	PubMed, Scopus, CINAHL, PsycINFO	48	General population, HCW	Demographics, social factors, vaccination beliefs and attitudes, vaccine-related perceptions, health-related perceptions, perceived barriers, vaccine recommendations
Bayou and Amare (35)	2022	Systematic review	31.4–92.33	PubMed, Google Scholar, Science Direct	21	Ethiopian	Age, sex, educational status, perceived susceptibility, perceived benefit, knowledge about COVID-19 vaccine, other socio-demographic factors
Belay et al. (36)	2022	Systematic review and meta-analysis	51.2	PubMed, Embase, Web of Science, Google Scholar, Ethiopian universities' research repository	14	Ethiopian	Having good knowledge, chronic disease, older age, secondary education and above
Bhattacharya et al. (37)	2022	Systematic review and meta-analysis	49	MEDLINE, Embase, CINAHL, PubMed	17	Pregnant women	High- income countries (-), participants with fewer than 12 years of education (-), multiparous women (-), COVID- 19 knowledge
Biswas et al. (38)	2021	Scoping review	28–86.1	Embase, PubMed, Google Scholar	82	General population	Vaccine efficacy, vaccine side effects, mistrust in healthcare, religious beliefs, trust in information sources, demographic factors (age, gender, education)
Chen et al. (39)	2022	Systematic review and meta-analysis	61.4	PubMed, Embase	29	Parent	Age of parents and guardians, access to scientific information and recommendations, routine and influenza vaccination behavior, willingness of parents and guardians to vaccinate themselves
Desye (40)	2022	Systematic review	21–95	PubMed, Science Direct, Web of Science, Google Scholar	33	HCW	Gender (male), age, profession (medical doctors), previous influenza vaccination
Galanis et al. (41)	2021	Systematic review and meta-analysis	63.5	PubMed, MEDLINE, Scopus, Web of Science, ProQuest, CINAHL, medRxiv	24	HCW	Gender (male), age (older), white people, HCWs, higher education level, comorbidity among HCWs, vaccination against flu during previous season, stronger vaccine confidence, positive attitude toward COVID-19 vaccine, fear about COVID-19, individual perceived risk about COVID-19, contact with suspected or confirmed COVID-19 patients
Galanis et al. (42)	2022a	Systematic review and meta-analysis	79	Scopus, Web of Science, Medline, PubMed, ProQuest, CINAHL, medrxiv	14	General population, HCW	Older age, flu vaccination in the previous season, confidence in COVID-19 vaccination, adverse reactions and discomfort experienced after previous COVID-19 vaccine doses (-), concerns for serious adverse reactions to booster doses (-)
Galanis et al. (43)	2022b	Systematic review and meta-analysis	60.1	Scopus, Web of Science, Medline, PubMed, CINAHL, medrxiv	44	Parent	Fathers, older age of parents, higher income, higher levels of perceived threat from the COVID-19, positive attitudes toward vaccination (e.g. children's complete vaccination history, history of children's and parents' vaccination against influenza, confidence in vaccines and COVID-19 vaccines, COVID-19 vaccine uptake among parents)
Geng et al. (44)	2022	Systematic review and meta-analysis	69	PubMed, Web of Science, Cochrane Library, CNKI	34	College student	Knowledge, trust conception, social behavior, information sources, country
Halemani et al. (45)	2022	Systematic review and meta-analysis	54	PubMed, Clinical key, Google Scholar, Cochrane Library, CINAHL	24	Pregnant women	Risks of infections, comorbidities, adverse effects (-), safety concerns (-)
Hajure et al. (46)	2021	Systematic review		Google Scholar, Science Direct, PubMed	24	HCW	Age, sex, profession, concerns about the safety of vaccines and fear of COVID-19, trust in the accuracy of the measures taken by the government, flu vaccination during the previous season, comorbid chronic illness, history of recommendation, depression symptoms
Januszek et al. (47)	2021	Systematic review	29.7–77.4	PubMed	9	Pregnant women	Trust in the importance and effectiveness of vaccine, explicit communication about the safety of COVID-19 vaccines, acceptance of other vaccinations (e.g., influenza), belief in the importance of vaccines/mass vaccination, anxiety about COVID-19, trust in public health agencies/health science, compliance to mask guidelines, older age, higher education, socioeconomic status
Joshi et al. (48)	2021	Scoping review	72	PubMed	22	General population	Socio-demographic variables (gender, age, education, occupation), trust in authorities, risk perception of COVID-19 infection, vaccine efficacy, current or previous influenza vaccination, vaccine safety, study period
Kalu et al. (37)	2022	Mapping review		PubMed, Ovid, Embase, CINAHL, PsychINFO	68	African countries	Sociodemographic factors; knowledge, attitude, and belief-related factors; COVID-19 vaccine efficacy and safety concern factors; trust in government and public health authorities
Kamal et al. (49)	2021	Rapid review		Web of Science, Ovid, Scopus, PsychINFO, Google Scholar	21	Minority ethnic groups in the UK	Inclusive communications which address vaccine concerns via trusted communicators, increased visibility of minority ethnic groups in the media, pre-existing mistrust of formal services (-), lack of information about the vaccine's safety (-), misinformation (-), inaccessible communications (-), logistical issues (-)
Kazeminia et al. (50)	2022	Systematic review and meta-analysis	63.9	PubMed, Embase, Scopus, Web of Science, Google Scholar	98	Not specific	Older adult and young people, medical staff, employees, education level, socioeconomic status, trust in vaccine, positive vaccination history
Kukreti et al. (51)	2022	Systematic review and meta-analysis	60.1	Cochrane Library, Medline, Embase, Registers	19	General population	COVID-19 cases per million population, deaths per million population, WHO regions of the world
Li et al. (52)	2021	Rapid systematic review	27.7–77.3	PubMed, Embase, Science Direct, Web of Science, China National Knowledge Infrastructure, VIP, Wanfang Data	13	HCW	Male, older age, physicians, previous influenza vaccination, self- perceived risk, concerns for safety (-), efficacy and effectiveness (-), distrust of the government (-)
Limbu et al. (21)	2022	Systematic review and meta-analysis	73.19	PubMed, CINAHL, Web of Science, and Google	43	General population	Attitude, subjective norms, perceived behavioral control, self-efficacy, region (continent), sample population
Lin et al. (53)	2021	Rapid review	50	PubMed, Embase, PsycINFO	126	General population	Perceived risk, concerns over vaccine safety and effectiveness, doctors' recommendations, inoculation history, political party orientation, perceived political interference
Lin et al. (54)	2022	Systematic review and meta-analysis	Practitioners (81.1%), students (60.5%).	Google Scholar, PubMed, Web of Science, Science Direct, Cochrane Library, EBSCO, LILACS, Open Gray	10	Dental student, dental practitioner	Dental practitioners from middle East and high-income countries
Luo et al. (55)	2021	Systematic review and meta-analysis	51	4 English databases (PubMed, Embase, Web of Science, the Cochrane Library) and 4 Chinese databases (CNKI, VIP, Wanfang Database, CBM)	9	HCW	Male, aged 30 years or older, having a history of prior influenza vaccination
Mahmud et al. (56)	2022	Systematic review and meta-analysis	62.79	PubMed, Medline, Web of Science, Google Scholar	79	General population, HCW	Pre- to post-pandemic (-), region (South-East Asia), region (Africa) (-)
Mose et al. (57)	2022	Systematic review and meta-analysis	51.64	PubMed, Scopus, Google Scholar, African Journals Online, Web of Science	12	Ethiopian	Male, secondary and above educational status, knowledge, positive attitude
Nehal et al. (58)	2021	Systematic review and meta-analysis	66.01	PubMed	63	General population	Age, gender, education, attitudes and perceptions about vaccines
Nindrea et al. (59)	2021	Systematic review and meta-analysis		ProQuest, PubMed, EBSCO	24	General population	Female, older age, high income, high education, high level of knowledge, encountered with COVID-19, fear about COVID-19, perceived benefits, flu vaccine during the previous season, HCWs, male, married, perceived risk, trust in health system, chronic diseases
Norhayati et al. (60)	2022	Systematic review and meta-analysis	61	PubMed	172	Not specific	Regions, population, gender, vaccine effectiveness, survey time, continent, HCWs, vaccine effectiveness, during the first survey
Olu-Abiodun et al. (61)	2022	Rapid review	20–58.2	PubMed, Web of Science, Cochrane Library, Embase	10	Nigerian	Propaganda (-), adverse effect concerns (-), conspiracy theories (-)
Parthasarathi et al. (62)	2022	Systematic review and meta-analysis	70	PubMed, MEDLINE, Scopus	35	General population	Study period (-), female gender (-), rural residence (-), lower income (-), lower formal education (-)
Patwary et al. (63)	2022	Rapid review	58.5	PubMed, Scopus, Web of Science	36	Low-and lower-middle income countries	Male, perceiving risk of COVID-19 infection
Popa et al. (64)	2022	Literature review		PubMed, Google Scholar	44	Eastern European countries	Public confidence in the vaccines' safety and efficacy, vaccine literacy, public trust in the government and the medical system
Prabani et al. (65)	2022	Systematic review and meta-analysis	59	PubMed, Science Direct, the Cochran	29	Patients with cancer	Early cancer stages (stages I and II), good compliance with prior influenza vaccinations
Ripp and Roer (66)	2022	Rapid review		COVID-19 Data Portal, APA PsycArticles, Psychology and Behavioral Sciences, Scopus, PubMed	10	General population	Belief in COVID-19-related conspiracy narratives
Robinson et al. (67)	2021	Systematic review and meta-analysis		PubMed, Scopus, pre-printer servers	28		Female (-), younger (-), lower income or education level (-), belonging to an ethnic minority group (-)
Roy et al. (68)	2022	Systematic review		PubMed, Elsevier, Science Direct, Scopus	47	General population	Safety, efficacy, side effects, conspiracy beliefs (Asian countries), trust, social influence (Europe), information sufficiency, political roles, vaccine-mandates (United States)
Sahile et al. (69)	2022	Systematic review and meta-analysis	57.8	Google Scholar, Web of Science, Science Direct, Hinari, Embase, PubMed	18	Ethiopian	Region, country
Shakeel et al. (70)	2022	Systematic review		PubMed, Web of Science, IEEE Xplore, Science Direct	81	General population	Country, low levels of education and awareness, inefficient efforts and initiatives by the government
Shamshirsaz et al. (71)	2022	Systematic review and meta-analysis	47	PubMed, Scopus, archive/pre-print servers	12	Pregnant women	Uptake of other vaccines (influenza and/or TdaP) during pregnancy
Shui et al. (72)	2022	Systematic review and meta-analysis	78	PubMed, Embase, The Cochrane Library, Web of Science, CNKI, Wanfang Database, CBM, VIP	18	HCW	Survey time, male, educational level (-), nurses (-) vs. doctors and other HCWs, regions, HCWs who participated in quarantine or had been in contact with confirmed cases
Terry et al. (73)	2022	Systematic review and meta-analysis		Medline, Embase, CINAHL, PsycINFO, PsycARTICLES, Sociological Abstracts, Applied Social Sciences Index and Abstracts	23	General population	Greater perceived risk of COVID-19, lower of perceived vaccine harm, higher education, higher household income, older age, ethnicity, male
Wake (74)	2021	Systematic review	27.7–91.3	PubMed/Medline, HINARI, Embase, Google Scholar, Web of Science, Scopus, African journals, Google for gray literature	45	General population	Age, education, gender, income, residency, occupation, marital status, ethnicity, perceived risk, trust in healthcare system, health insurance, norms, attitude toward vaccine, perceived benefit, perceived barriers, self-efficacy, vaccination status, history of COVID-19 infection, perceived efficacy, recommended for vaccination, political leaning, perceived severity, vaccine safety concern, fear about COVID-19, cues to action, presence of chronic disease, confidence, vaccine hesitancy, complacency
Wang et al. (75)	2021	Systematic review and meta-analysis	73.31	PubMed, Web of Science, Cochrane Library, Embase	38	General population, HCW	Gender, educational level, influenza vaccination history, trust in the government, protecting oneself or others, concerns about side effects and safety (-)
Wang et al. (76)	2022	Systematic review and meta-analysis	67.8	PubMed, Embase, Web of Science, EBSCO	519	General population	Pregnant/lactating women (-), country, study period (-), aged < 60 years (-), Black people (-), lower education (-), lower income (-)
Willems et al. (77)	2022	Scoping review	27.7–92	CINAHL, APA PsycArticles, APA PsycInfo, Web of Science, Semantic Scholar, Prospero, Outbreak Science, Cochrane, Scopus	26	HCW	Profession, age, gender, education, income, ethnicity, geographical, political orientation, past vaccine behavior, comorbidities, mental well-being, COVID self-history, COVID family history
Zintel et al. (78)	2022	Systematic review and meta-analysis		PubMed, Web of Science, PsycInfo	46	General population	Women (-), HCWs (-)

HCW, Healthcare worker.

Table 4 presents vaccination intention rates reported by the included reviews. The average rate of COVID-19 vaccination intention was 56.97% (SD = 20.05), ranging from 46% (29) to 78% (23). The highest average vaccination intention rate was reported by systematic reviews with meta-analyses (62.53%), followed by rapid reviews (54.25%). Vaccination acceptance rate differed by population type (*F*(4, 24) = 3.845, *p* ≤ 0.05). Average vaccination intention rate was highest among general populations (68.36%), followed by healthcare workers (64.8%) and parents (60.75%). Vaccine acceptance was lowest among pregnant women (50.87%).

### 3.2. Factors influencing COVID-19 vaccination intention

#### 3.2.1. Socio-demographic

Table 5 shows that the most frequent socio-demographic predictors of CVI were gender, age, education, income, occupation, ethnicity, and marital status. Gender predicted CVI in almost one-half of the included reviews (27/55). Several reviews indicated that males were more likely to accept COVID vaccines than females (30, 40, 41, 52, 55, 57, 60, 63, 72, 73). Other reviews reported that women were less willing to get vaccinated against COVID-19 (37, 62, 67, 76, 79). Similarly, Wang et al. (76) reported that pregnant/lactating women have the lowest vaccination intention.

**Table 5:** Factors influencing COVID-19 vaccination intention.

**Themes**	**Factors influencing COVID-19 vaccination intention**
1. Sociodemographic (i) Gender	Positive effect • Male (30, 40, 41, 52, 55, 57, 60, 63, 72, 73) Negative effect • Female (62, 67, 76, 79) • Pregnant/lactating women (76) • Multiparous women (37) Direction of effect not specified • Gender (32, 35, 38, 46, 48, 58, 60, 74, 75, 77)
(ii) Age	Positive effect•Older age (36, 41–43, 47, 59, 73) • Young age groups (20–40 years old) and the older adult (over 60 years old) compared to other age groups (50) Negative effect•Younger (67) Direction of effect not specified•Age (32, 35, 38, 40, 46, 48, 58, 74, 77)
(iii) Education	Positive effect•Higher education (41, 47, 50, 57, 59, 73) • Secondary education and above (30, 36) Negative effect•Lower education (37, 62, 67, 70, 76) • Higher educational level (72) Direction of effect not specified•Education (32, 35, 38, 48, 58, 74, 75, 77)
(iv) Income	Positive effect•Higher income (43, 54, 59, 73) Negative effect • Lower income (62, 67, 76) Direction of effect not specified•Income (32, 74, 77)
(v) Occupation/profession	Positive effect•Healthcare workers (41, 59, 60, 74) • Physicians (40, 41, 52, 54) • Medical staff (50) • Healthcare workers vs. healthcare students (29) • Healthcare workers who participated in quarantine or had been in contact with confirmed cases (72) • employees (50) Negative effect•Healthcare workers (79) • Nurses vs. doctors and other healthcare workers (52, 72) • Healthcare workers vs. general population (31) Direction of effect not specified•Occupation (48, 74) • Profession (46, 77)
(vi) Ethnicity	Positive effect•White people (41, 49, 73) Negative effect • Black people (76) • Belonging to an ethnic minority group (49, 67) Direction of effect not specified•Ethnicity (74, 77)
(vii) Marital status	Positive effect•Married (59, 74)
2. Geographical	Positive effect•Continent (Asia) (44, 56) • Region (middle East) (54) • Region (South America) (31) • WHO regions of the world (51) • High-income countries (54) Negative effect • Continent (Europe) (56) • Region (Africa) (56) • Region (Middle East (31) • Rural residence (62) • High-income countries (37) Direction of effect not specified•Geographical (77) • Continent (21, 60) • Country (69, 70, 76) • Residency (74) • Regions (29, 31, 38, 60, 72)
3. Social factor	Positive effect•Social influence (Europe) (68) • COVID-19-related prosocial behaviors (44) • Social factors affecting thoughts/attitude in social contexts in general situations (32)
4. Political factor	Positive effect•Political leaning (Liberal or moderate) (74) • Political party orientation (53, 77) • Political roles (68) Negative effect • Political issues (23) • Perceived political interference (53)
5. Government role	Positive effect•Vaccine-mandates (United States) (68, 74) Negative effect•Inefficient efforts and initiatives by the government (70)
6. Study time	Positive effect•Survey year (72) • Survey month (33) Negative effect•CVI declined from 2020 to 2021 (76) • CVI declined in the second half of the study period when compared to the first half (62) • CVI declined from March 2020 to September 2020 (48) • CVI declined pre- to post-pandemic (56) • CVI declined from first survey to second survey (60)
7. Attitude	Positive effect•Attitude toward vaccine (21, 30, 32, 34, 41, 57, 58, 74, 80) • Attitudes toward vaccination (34, 43)
8. Perceived severity	Positive effect•Lower level of perceived vaccine harms (73, 76) Negative effect•Perceived severity of COVID-19 infection (76) • Concerns for adverse reactions to COVID-19 vaccine (23, 42, 45, 61) • Concerns about side effects and safety (29, 38, 45, 52, 53, 68, 75, 80)
9. Perceived susceptibility	Positive effect•perceiving risk/susceptibility of COVID-19 infection (1, 35, 41, 43, 45, 48, 52, 53, 59, 63, 73, 74) • fear about COVID-19 (41, 46, 59) • anxiety about COVID-19 (27)
10. Perceived benefits	Positive effect•Perceived benefit of COVID-19 vaccine (23, 35, 59, 74) • Perceived efficacy of the COVID-19 vaccine (29, 38, 48, 60, 68, 74, 80) • Public confidence in the vaccines' efficacy (29, 48, 64, 68) Negative effect • Concerns about efficacy and effectiveness of COVID-19 vaccine (52)
11. Perceived barriers	Negative effect•Financial barriers (34, 74) • Shortage of vaccine (79) • Logistical issues (49)
12. Self-efficacy and perceived behavioral control	Positive effect•Confidence in their ability to receive COVID vaccine (21, 41, 42, 74) • Perceived behavioral control (21) Negative effect•Low confidence in the health system (1)
13. Norms	Positive effect•Subjective norms (21) • Social norms (74)
14. Trust	Positive effect•Trust in vaccine (50, 68) • Trust in the vaccine effectiveness (28) • Trust in public health agencies/health science (47) • Trust in healthcare system (59, 74) • Trust in medical system (64) • Trust in government and public health authorities (44, 48, 64, 75, 80) • Trust in the accuracy of the measures taken by the government (46) • Trust in information sources (38, 44) Negative effect • Mistrust in healthcare system (38) • Distrust of the government and healthcare system (23, 53) Lack of social trust (29)
15. Conspiracy theory, propaganda, and misinformation	Negative effect•Anti-vaccine conspiracy theories and beliefs (61, 66, 68) • Propaganda (59) • Misinformation or negative information (1, 23, 49)
16. Knowledge	Positive effect•Knowledge about COVID-19 vaccine (30, 35, 57, 59, 64, 80) • Knowledge about COVID-19 (32, 44)
17. Information and Communication	Positive effect•Information sufficiency (68) • Inclusive communications which address vaccine concerns via trusted communicators (49) • Increased visibility of minority ethnic groups in the media (49)
	•Explicit communication about the safety of COVID-19 vaccines for pregnant women (47) • Trusted information sources (44) • Access to scientific information from public health authorities and physicians (39) Negative effect • Lack of information about the vaccine's safety (29, 49) • Inaccessible communications (49)
18. Recommendation for vaccination	Positive effect•recommended for vaccination by others (34, 39, 53, 74) • recommended for vaccination to others (46)
19. Vaccination history	Positive effect•Influenza vaccination history (39–43, 48, 52, 55, 59, 65, 71, 75) • Inoculation history (50, 53, 77) • Up-to-date on vaccinations (74) • Receiving any vaccine in the past 5 years (74)
20. History of COVID-19 infection	Positive effect•COVID-19 self-history (29, 30, 59, 74, 77) • COVID-19 family history (77) Negative effect • Previous COVID-19 infection (28)
21. Health status and well-being	Positive effect•Having chronic diseases (28, 36, 59, 74) • Comorbidities (41, 45, 46, 77) • Early cancer stages (stages I and II) (65) • Depression symptoms in the past week (46) • Mental well-being (77)
22. Other factors	Positive effect•Contact with suspected or confirmed COVID-19 patients (41) • Health insurance (74) • Religious beliefs (38) • Cues to action (74)

Several reviews reported that older people were more likely to accept COVID vaccines (36, 41–43, 47, 52, 59, 73). On the other hand, younger individuals were less likely to get vaccinated against COVDI-19 (67). However, Kazeminia et al. (50) revealed mixed findings; young age groups (20–40 years old) and the older adult population (over 60 years old) demonstrated more CVI than other age groups.

Twenty-one reviews reported education attainment as a significant predictor of CVI, but findings are inconclusive. A higher level of educational attainment was positively associated with a higher level of CVI in eight reviews (30, 36, 41, 47, 50, 57, 59, 73). Conversely, a lower level of education was negatively associated with CVI in five studies (37, 62, 67, 70, 76). On the contrary, Shui et al. (72) reported the opposite in which the willingness of healthcare workers to vaccinate against COVID-19 declined with higher levels of education.

Ethnicity was a significant predictor of CVI in six reviews. For example, a higher level of COVID-19 vaccine acceptance was found in White people (41, 49, 73). Conversely, Black people (76) and minorities (67) demonstrated lower CVI. Similarly, a study showed that ethnic minorities had significantly lower vaccine uptake compared to White British groups (49).

In regard to marital status, married individuals were more likely to accept COVID vaccines (59, 74). When it comes to income, higher income was positively associated with a higher level of CVI (43, 54, 59, 73). On the other hand, people with lower incomes had lower vaccine acceptance (62, 67, 76).

Contradictory evidence was reported on the association between occupation and CVI. For example, eleven reviews reported that healthcare workers such as dental practitioners (54) were more likely to accept COVID vaccines (29, 40, 41, 50, 52, 59, 60, 72, 74). However, two reviews found the opposite (31, 79). Therefore, the impact of occupation on healthcare workers' intentions to get vaccinated has not yet been confirmed (55).

#### 3.2.2. Geographical factors

Geographical factors such as region, country, continent, and residency were found to be associated with CVI, but the findings are mixed. For example, a higher COVID-19 vaccine acceptance rate was reported in South-East Asia (44, 56), the Middle East (54), high-income countries (54), South America (31), and WHO regions of the world (51). On the contrary, other studies reported lower COVID-19 vaccine acceptance in high-income countries (37), Europe (44), Africa (56), the Middle East (31), and rural areas (62).

#### 3.2.3. Social factor

Roy et al. (68) highlighted the role of social influence on CVI. The authors revealed that opinions from friends, family, and social networks significantly affected CVI, especially in Europe and the United States. Geng et al. (44) found that COVID-19-related prosocial behaviors (e.g., donating resources and providing help to those affected by COVID-19) were positively associated with increased CVI. Social factors that affected people's thoughts or attitudes in social contexts in general situations (e.g., social density, prosocial concern, communication and media, social solidarity) positively impacted vaccination intention against COVID-19 (34).

#### 3.2.4. Political factor

Major political factors that influenced CVI included political leaning (being moderate or liberal) (74), political party orientation (54, 77), and political roles (68). Other factors that had negative associations with CVI were political issues (i.e., purchase of vaccine batches, quarantine isolation measures, vaccination process implementation) (23) and perceived political interference (53).

#### 3.2.5. Government role

Vaccine mandates in the United States (68) and believing in mandatory COVID-19 vaccination (74) were significant determinants of COVID-19 vaccine acceptance. However, inefficient efforts and initiatives by the government had an adverse effect on CVI (70).

#### 3.2.6. Study timeline

Vaccination intentions varied by survey time (72). For example, most reviews reported that the average COVID-19 acceptance rate declined over time. In addition, the acceptance rate declined in the second survey period compared to the first survey period (60), in the second half of the study period when compared to the first half (62), from March 2020 (86%) to September 2020 (72%) (48) and from pre-pandemic period to post-pandemic period (56). Furthermore, the acceptance rate declined globally from December 2020 to late 2021 (76). On the contrary, one study reported that the pooled acceptance rate of COVID-19 vaccine among healthcare workers in China was higher in 2021 than in 2020 (72).

#### 3.2.7. Attitude

Attitudes toward vaccines (21, 30, 32, 34, 41, 57, 58, 74, 80) and attitudes toward vaccination (34, 43) were positively associated with CVI. Moreover, the attitude had a significant influence in Asia, Europe, and Oceania, especially among adults, parents, and patients (21).

#### 3.2.8. Perceived severity

Several studies identified the perceived severity of COVID-19 infection (74), concerns for adverse reactions to COVID-19 vaccine (23, 41, 45, 61), and concerns about side effects and safety of COVID vaccines (29, 38, 45, 52, 53, 68, 75, 80) as the common predictors of COVID-19 vaccine acceptance. In addition, Halemani et al. (45) stated that adverse effects were the top indicators for rejecting the COVID vaccine. On the other hand, a lower level of perceived vaccine harms (73, 74) was positively related to CVI.

#### 3.2.9. Perceived susceptibility

The association between perceived susceptibility (perceived risk of contracting COVID-19) and CVI was reported in 17 studies. Perceiving susceptibility to COVID-19 infection (1, 35, 41, 43, 45, 48, 52, 53, 59, 63, 73, 74), fear about COVID-19 (41, 46, 59) and anxiety about COVID-19 (47) were key drivers of CVI. In addition, the risks of infections were one of the main reasons for accepting the COVID vaccine in pregnant women (45).

#### 3.2.10. Perceived benefits

Our study also shows that the perceived benefit of the COVID-19 vaccine (35, 59, 74, 75), perceived efficacy of the COVID-19 vaccine (29, 38, 47, 48, 60, 68, 74, 80), and public confidence in the vaccines' efficacy (29, 48, 64, 68) positively influenced CVI. Similarly, Januszek et al. (47) found the perceived effectiveness of the vaccine as a strong factor co-existing with the acceptance of the COVID-19 vaccination during pregnancy. On the other hand, concerns about the efficacy and effectiveness of the COVID-19 vaccine negatively impacted CVI in healthcare workers (52).

#### 3.2.11. Perceived barriers

A few reviews reported that perceived vaccination barriers such as shortage of vaccines (79), logistical issues (49), and financial barriers (34, 74) significantly impaired vaccination intention against COVID-19.

#### 3.2.12. Self-efficacy and perceived behavioral control

People's confidence in their ability to receive the COVID-19 vaccine (21, 41, 42, 74) influenced COVID-19 vaccine acceptance. For example, low confidence in the health system reduced CVI (1). In a systematic review and meta-analysis, perceived behavioral control (i.e., whether the ability to get the vaccine is within an individual's control) was found as one of the dominant drivers of vaccination intention, especially among African patients (21).

#### 3.2.13. Norms

Limbu et al. (21) showed that subjective norms (i.e., the perception that a family member would support them in having a COVID-19 vaccination) had a dominant effect on CVI in Asia and Oceania, especially among parents and patients. Another study found social norms (i.e., whether valued others support getting a vaccine) as an influential predictor of behavioral intention to vaccinate against COVID-19 (72).

#### 3.2.14. Trust

Numerous reviews reported trust as a crucial determinant of CVI. Trust-related factors that affected CVI included trust in the vaccine (50, 68), trust in the vaccine effectiveness (28), trust in public health agencies/health science (47), trust in healthcare system (59, 74), trust in medical system (64), trust in government and public health authorities (44, 48, 64, 75, 80), trust in the accuracy of the measures taken by the government (46), and trust in information sources (38, 44). On the contrary, people's mistrust of the healthcare system (38) and distrust of the government and healthcare system (23, 53) decreased CVI. A low acceptance of the COVID-19 vaccine was impacted by the lack of social trust (i.e., insufficient trust in the vaccine's source, lack of trust from the manufacturers, and lack of trust from governments) (29).

#### 3.2.15. Conspiracy theory, propaganda, and misinformation

Some studies found that anti-vaccine conspiracy theories and beliefs (61, 66, 68), propaganda (61), and misinformation or negative information (1, 23, 49) significantly impaired people's intentions to get vaccinated against COVID-19.

#### 3.2.16. Knowledge

A higher level of knowledge about COVID-19 vaccines was positively associated with a higher level of vaccination intention (30, 35, 57, 59, 64, 80). Likewise, knowledge about COVID-19 significantly increased people's vaccination intentions (32, 44).

#### 3.2.17. Information and communication

Information- and communication-related factors such as information sufficiency (68), inclusive communications which address vaccine concerns via trusted communicators (49), increased visibility of minority ethnic groups in the media (49), explicit communication about the safety of COVID-19 vaccines for pregnant women (47), trusted information sources (44), and access to scientific information from public health authorities and physicians (39) were strong drivers of CVI. On the contrary, lack of information about the vaccine's safety (29, 49) and inaccessible communications (49) were significant barriers to CVI.

#### 3.2.18. Recommendation for vaccination

Some reviews indicated that people's vaccination intentions were influenced by the recommendations from public health authorities and physicians (34, 39, 53, 74). In addition, people's tendencies to recommend vaccination to others were positively associated with CVI (46).

#### 3.2.19. Vaccination history

Past vaccine behavior was one of the most powerful predictors of the willingness to be vaccinated against COVID-19 (77). Inoculation history (50, 53), including influenza vaccination history (39–43, 46, 48, 52, 55, 59, 65, 71, 75), up-to-date vaccinations (74), and receiving any vaccine in the past 5 years (74) were positively associated with a higher level of CVI.

#### 3.2.20. History COVID-19 infection

Some reviews reported that prior COVID-19 infection (29, 30, 59, 74, 77) and family history of COVID-19 infection (77) were significant determinants of CVI. Conversely, one study showed that previous COVID-19 infection was associated with a lower intention to have the booster dose (28).

#### 3.2.21. Health status and well-being

Individuals with chronic diseases (28, 36, 59, 74), such as comorbidities (41, 45, 46, 77) and early cancer stages (65), were more likely to get vaccinated against COVID-19. Similarly, mental well-being was positively associated with increased CVI (77). However, one study reported that depression symptoms strengthened the willingness to get vaccinated (46).

#### 3.2.22. Other factors

Other common determinants affecting vaccination intention included health insurance (74), religious beliefs (38), and cues to action (74).

## 4. Discussion

Mass vaccination is the most successful and cost-effective public health intervention to overcome a pandemic like COVID-19, as it has significantly contributed to improving global health by reducing mortality caused due to many infectious diseases (81, 82). However, despite the availability of vaccines and the mass global drive for vaccination, many people remain hesitant to be vaccinated, are less inclined to receive booster shots, or are even less likely to vaccinate their offspring (21). As a result, several countries, including some African countries, have low vaccination rates or yet to achieve herd immunity (81). There are several barriers to achieving the desired goal of vaccination coverage. According to Alam et al. (83), to achieve a higher coverage of the vaccines and to attain herd immunity, it is essential to elicit a positive attitude toward COVID-19 vaccines amongst individuals and populations. Furthermore, it is imperative to identify the causes of refusal/hesitancy and accordingly develop appropriate interventions. Hence, this meta-review was carried out to provide a comprehensive understanding of the factors influencing COVID-19 vaccination intention. The results of this study will be helpful to the agencies involved in vaccination and the prevention and control of pandemics around the globe.

This meta-review found a moderate COVID-19 vaccination acceptance rate of 56.97% globally. Vaccine acceptance was higher among healthcare workers, parents, and seniors, but some populations, such as young people and women, were more hesitant to receive primary series or booster doses. These results indicate that there is a need to improve vaccine coverage among specific populations (76). Thus, targeted communication and intervention approaches can be used to increase vaccine uptake among such populations.

We identified twenty-one main clusters of predictors that influenced COVID-19 vaccination acceptance, including socio-demographic, geographical, political, attitude, perception, norm, trust, knowledge, and vaccine-related factors. These results indicate that COVID-19 vaccination acceptance is a complex process and is affected by numerous multifaceted factors.

The most frequent socio-demographic predictors of vaccine acceptance were gender, age, education, income, and occupation. All systematic reviews that synthesized evidence on gender effect concluded that females were more likely to be vaccine-hesitant. In terms of age, younger individuals were associated with being less likely to intend to vaccinate. In addition, several studies reported that ethnic minorities and individuals with a lower level of income and education had a lower level of intention to get vaccinated against COVID-19. Thus, these results clearly suggest that it is important to understand why different socio-demographic groups, such as females, young individuals, and low-income populations, demonstrate lower intentions to vaccinate against COVID-19 and develop targeted information campaigns and interventions that could enhance their vaccination intentions (62, 67). However, such campaigns should focus on improving awareness of the efficacy of COVID-19 vaccines (36).

Results also show that COVID-19 vaccine acceptance varies by geographic location. This variability was evident in different countries and regions of the world. However, the evidence is contradictory and inconclusive. For example, some reviews reported higher vaccine acceptance rates in South-East Asia, the Middle East, high-income countries, South America, and WHO regions of the world (31, 44, 51, 54, 56). On the contrary, other reviews reported lower vaccine acceptance in high-income countries, Europe, Africa, the Middle East, and rural areas (31, 37, 44, 56, 62). More research is needed to shed light on regional disparities in COVID-19 vaccine acceptance (56). Moreover, reasons for not accepting COVID-19 vaccines should be investigated across different geographic locations (region, country, residency), and targeted measures should be taken into account to improve COVID-19 vaccine acceptance according to their local contexts (76).

Our results show that social factors were influential drivers of individuals' vaccination willingness. Opinions provided by friends, family, and social networks had significant effects on vaccine acceptance. In addition, COVID-19-related prosocial behaviors (e.g., donating resources and providing help to those affected by COVID-19) and social factors that affected people's thoughts or attitudes in social contexts in general situations (e.g., social density, prosocial concern, communication and media, social solidarity) positively impacted vaccination acceptance. Moreover, recommendations from public health authorities and healthcare providers influenced people's vaccination intentions. Moreover, individuals' vaccination intentions were influenced by the recommendations from public health authorities and healthcare providers. Thus, effective vaccination communication strategies may include encouragement from loved ones and trusted figures, such as family, friends, physicians and religious leaders (84).

The decision to accept COVID-19 vaccination was also influenced by political factors (e.g., political leaning, political roles, political interference) and government roles (e.g., vaccine mandates, government initiatives). Hence, government institutions should implement strategies that help to eliminate political barriers. In addition, COVID-19 vaccine mandates for healthcare workers and other vulnerable populations (e.g., older adult and co-morbid individuals) and information dissemination and recommendations from trusted government officials and political leaders can be effective strategies in improving vaccination acceptance (68).

Individual factors, such as attitudes (e.g., attitudes toward vaccination and vaccines), perceptions, and beliefs, were dominant predictors of CVI. An effective attitude change strategy for COVID-19 vaccine uptake will benefit from focusing on populations with negative attitudes, especially among adults, parents, and patients in Asian, European, and Oceania countries (21). The results of the present study show that the most frequently demonstrated perceptions and beliefs that impacted vaccination intentions were perceived severity, perceived susceptibility, perceived benefits, perceived barriers, self-efficacy, and perceived behavioral control. Therefore, future public awareness and educational campaigns aimed at promoting COVID-19 vaccines should focus on these factors and consider using psychological theories such as the health belief model and theory of planned behavior as conceptual frameworks for designing stimuli and effective interventions (4, 21, 85). Such campaigns should highlight the potential risk of contracting COVID-19/risks of infections, the advantage of COVID-19 vaccines, and the efficacy of the COVID-19 vaccines. More importantly, further data and information on the safety and efficacy of vaccines should be provided with transparency (52). To enhance public confidence and uptake of COVID vaccines, addressing people's vaccine-related concerns, such as side effects and adverse reactions, is essential. It is also vital to address vaccination barriers, such as concerns associated with accessibility and cost of vaccines. However, integrated global efforts are required to overcome such barriers (56).

Numerous reviews included in our study reported distrust (e.g., lack of trust in vaccines, public health agencies, healthcare system, medical system, and information sources) and anti-vaccine conspiracy theories/beliefs (e.g., misinformation or negative information) as significant determinants of vaccination intention. Governments and other stakeholders engaged in the production, promotion, and distribution of vaccines should strengthen their credibility and convey trusted information through credible sources, focusing on transparency and restoring trust in health authorities. The spread of misinformation regarding vaccination and conspiracy theories should be taken very seriously and counterbalanced by targeted interventions and communication campaigns (53, 70, 77).

Some studies showed that a higher level of knowledge about COVID-19 vaccines and information- and communication-related factors (e.g., information sufficiency, inclusive communications, explicit communication about vaccine safety, and access to scientific information from public health authorities and healthcare providers) were strong drivers of vaccine acceptance. On the contrary, lack of information about the vaccine's safety and inaccessible communications were significant barriers. Therefore, governments and healthcare providers have to pay more attention to individuals and populations with lower levels of knowledge and implement policies to elevate their awareness about vaccination and vaccines through targeted education programs that are designed to increase their self-efficacy (21).

History of previous vaccination against COVID-19 or influenza was one of the most prevalent predictors of the willingness to be vaccinated against COVID-19. Moreover, a family history of COVID-19 infection was associated with a higher intention to have additional doses. Individuals with poor health (e.g., chronic diseases, comorbidities) were more likely to get vaccinated against COVID-19. Thus, these factors should be taken into account when developing interventions aimed at decreasing COVID-19 vaccine hesitancy.

This meta-review has several limitations; thus, the significance of these findings should be interpreted with caution. For example, we searched only four databases to locate systematic reviews; thus, some relevant studies might have been overlooked. In addition, systematic reviews included in this meta-review varied in terms of study populations and countries, which might have contributed to their inconsistent findings. Finally, we excluded non-English systematic reviews, which may limit the scope and validity of our results or may present publication bias.

This meta-review identified several important areas for future research: (1) several studies included in this review reported mixed findings, which warrants future research. Further studies are needed to shed light on inconclusive evidence, especially in regard to the role of gender, education, occupation, and geographic location; (2) a micro-level study should be conducted to understand minute cultural issues of COVID-19 vaccine hesitancy and acceptance; (3) this meta-review shows that vaccine acceptance was found lower among young individuals and women, hence future primary studies could investigate the reasons for their unwillingness to get vaccinated; (4) social and mass media have a pivotal role in promoting or making rumors against vaccines. Thus, future studies should explore deeper insights into the role of social media as a promoter or a barrier to vaccination campaigns; and (5) future research is needed to examine the impact of social capital (bonding, bridging, and linking) and a reference group (a person or group of people that significantly influences an individual's behavior) in influencing vaccination intention.

## 5. Conclusion

This meta-review reveals that there are wide disparities in vaccine acceptance across the globe, and several factors (e.g., psychological, demographic, geographical, political, and social) affect individuals' decision to accept a COVID-19 vaccine. A holistic educational approach to improve confidence in COVID-19 vaccines and multifaceted interventions may be effective for improving vaccination intention against COVID-19. However, a country- and population-specific strategy at amicro-level is required for a successful mass vaccination drive and manage the COVID-19.

## Data availability statement

The original contributions presented in the study are included in the article/supplementary material, further inquiries can be directed to the corresponding author.

## Author contributions

All authors listed have made a substantial, direct, and intellectual contribution to the work and approved it for publication.
